# Plant-Based Alternatives to Meat Products

**DOI:** 10.3390/foods14081396

**Published:** 2025-04-17

**Authors:** Claire Darizu Munialo, Vahid Baeghbali, Parag Acharya

**Affiliations:** 1 Harper Food Innovation, Harper Adams University, Newport TF10 8NB, UK; 2 Natural Resources Institute, Faculty of Engineering and Science, University of Greenwich, Central Avenue, Chatham Maritime, Kent ME4 4TB, UK; v.baeghbali@greenwich.ac.uk; 3 Bezos Centre for Sustainable Protein, University of Greenwich, Central Avenue, Chatham Maritime, Kent ME4 4TB, UK

**Keywords:** alternative proteins, functional properties, future foods, plant-based products, meat alternatives, sustainability

## Abstract

Animal proteins have been used in the formulation and production of food products for many centuries, which has mainly been attributed to their excellent functional properties. However, the rearing of animals has been associated with an increased emission of greenhouse gases that contributes to global warming and climate change. Consequently, there has been a drive toward using alternative proteins, such as those from plant origins, which have been found to be more sustainable. A climate-smart strategy to contribute toward a reduction in meat consumption has been the formulation of plant-based meat analogues. The lower acceptance of these meat substitutes is mainly attributed to their sensorial, nutritional, and textural properties, which fail to resemble conventional meat. As such, there is a knowledge gap in understanding key aspects that come into play while formulating meat alternatives from plant sources by deciphering the link between the techno-functional attributes of protein and the various quality attributes of these food products. Therefore, this review aims to discuss the technical advances that have been made when it comes to plant-based meat substitutes that could drive consumer acceptance. There is also a huge impetus to diversify plant protein usage in meat analogues beyond soy and pea, which requires the applications of underutilised plant proteins to overcome their functional and organoleptic shortcomings, as well as the techno-economic challenges that have also been addressed in this work. Additionally, the nutritional equivalency of plant-based meat alternatives is reviewed, and the ways in which these products have been fabricated are discussed to assess the opportunities and challenges that exist in current product formulations. Other key determinants, such as environmental sustainability factors, prospective supply chain issues, and the market adoptability of plant-based meat alternatives, are also discussed. This review emphasises the fact that interlinking technical challenges with consumer insights and socioeconomic perspectives for protein transition is critical to ensure that innovations successfully land in the market.

## 1. Introduction

The continued increase in the world’s population has a concomitant impact on food production and the demand for adequate nutrition. Animal proteins have been used for many centuries, as the conventional food proteins in the food industry, to formulate and produce a variety of food products; this is mainly attributed to their functional properties, which include emulsification, foaming, gelation, solubility, and water-holding and -binding capacities [1] However, the production and processing of protein from animal sources comes at the expense of environmental impacts due to the land and water requirements, pollution, and greenhouse gas (GHG) emissions associated with livestock production [2]. Therefore, as the global demand for protein is expected to further escalate with the increased growth of the human population, there is a need to sustainably produce proteins to feed ~9.6 billion people by 2050, which brings meat analogues/alternatives onto the centre stage for re-inventing a decarbonised food value chain [3,4,5]. Plant-based meat alternatives are considered an incipient solution to mitigate the GHG emissions associated with meat production and consumption and have the potential to substantially reduce agricultural land usage and to address the biodiversity goals of our food system [6]. Typically, meat analogues refer to emerging food products that try to mimic the taste, texture, and appearance of conventional meat. Aligning with global efforts to promote sustainable and equitable food system reforms, the landscape of meat analogues continues to evolve, with many researchers and the food industry working tirelessly to find sustainable alternatives to animal products [7,8,9,10,11].

The surging appeal for meat analogues is not only limited to vegetarians but also to non-vegetarians, popularly called flexitarians, who are opting for this partly to satisfy their meat cravings while reducing their overall meat consumption. Plant-based meat alternatives represent ~99% of today’s meat analogue market, and other sources, such as fermentation-derived or cultivated meat, are still under research or in the very early stage of commercialization [12]. As the process and product technologies for meat analogues continue to advance, there is a gradual change happening in the plant-based meat alternative sector as it moves from being niche to mainstream. Hence, there is a need to understand the use of alternative proteins in the formulation of plant-based meat alternatives and explore some of the methods that have been used in the fabrication of these alternatives. Therefore, this review aims to describe the landscape of current technological development across the ingredient choices, processing, and product formulations for plant-based meat alternatives, as well as to assess the concomitant environmental benefits, latest market trends, gaps in nutritional equivalency, and evolving consumer insights. Unlike the existing technical reviews on plant-based meat alternatives, this review has also emphasised the interlinking of knowledge gaps, prospective supply chain issues, and the influence of social dynamics on the forthcoming innovation challenges. As such, this work will contribute towards the existing knowledge on the possible applications of plant-based alternatives to meat products as sustainable, cheaper solutions.

## 2. Sources of Plant Proteins

Alternative protein sources, such as insects [13,14], fungi [15,16], cultivated meat [17,18], and plants [11,19], among others, have been researched, scoped in new food formulations, and commercialised as potential future foods that can replace proteins from animal sources [20]. However, given that myriad sources of protein have been investigated as sustainable alternatives to animal protein [7,8,9], this review mainly focuses on those of plant origin that have been researched and used in the formulation of meat alternatives.

**Figure 1:**
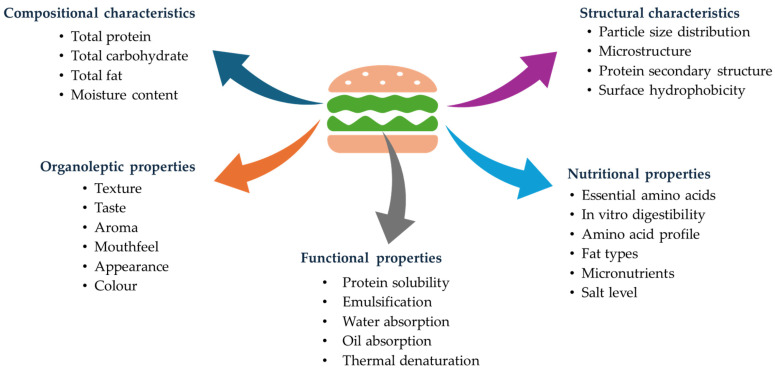
Key properties of plant-based meat alternatives, which are influenced by protein ingredient choices, processing, and product formulations.

Proteins of plant origin are considered to be cheaper and more sustainable than their animal counterparts, hence the reason for their continued use in the formulation of meat analogues. Even though plant-based protein alternatives are viewed as being more sustainable than those of animal origin, it is worth mentioning that when using proteins of plant origin, such as soy, pea, and wheat, among others, as main ingredient sources, the ingredient production and processing stages of product manufacturing can significantly contribute to an increased environmental impact. For example, a study showed the production cost of the bovine protein used for a 113 g burger formulation is almost 12 times more than that of the corresponding plant-based protein [21]. However, the economic and environmental costs for the preparation of isolates from proteins of plant origins may not be cheap due to varied protein extraction efficacies. Hence, the exploration of the functional properties of different plant protein sources as an integral part of future foods continues to be at the centre of food research to deliver improved end-product properties [22] that require less or minimal processing and formulation. For example, proteins of plant origin, such as soy [23,24,25], pea [26,27,28], lupine [29,30], faba bean [31,32], and quinoa [33,34,35], among others, have all been explored in terms of their techno-functional properties. Key determinants of the functional properties include the protein content, type, and composition [36,37]. Key properties of plant-based meat alternatives, which are influenced by protein ingredient choice, processing, and product formulation, are provided in Figure 1. The composition of meat alternatives, which includes the total protein, fat, moisture, and carbohydrate contents, can impact the nutritional aspects of the food product. Depending on the nature and type of ingredients used in the formulation, the structural, functional, nutritional, and organoleptic properties of the final product can be manipulated to result in the formulation of products that mimic animal meat. A summary of plant protein materials and other ingredients that are used for meat analogue applications is presented in Table 1.

Protein from soybeans has traditionally been used in the formulation of tofu and tempeh, whereas gluten from wheat is more associated with seitan [38]. Soy protein is also the most widely employed plant protein so far in meat alternative products available in the market. The use and selection of soy protein is, however, governed by the composition of the various fractions. For instance, the importance of selecting soy protein with a consistent glycinin (11S) and β-conglycinin (7S) 11S/7S ratio has been highlighted because such a protein composition (11S/7S ratio of 1.5:1 to 2:1) leads to high-quality meat analogue products [39]. For soy to be used in various applications, the conditions, such as the temperature, pH, and protein concentration, all need to be considered. The formation of protein aggregates can occur via the hydrophobic interaction of different proteins following heat treatment [40]. For example, the effect of heat-induced 7S and 11S aggregates in soy proteins has been researched. When 7S and 11S were heated individually, the unfolded 7S active sites in soluble aggregates were found to be limited. The unfolded 11S was shown to expose more active sites and to form larger aggregates, which was mainly due to the hydrophobic interaction [41]. However, the aggregation was terminated following the interaction between 7S and 11S during heating [42], and this was shown to have the potential to impact the functional characteristics of the protein aggregates in various matrices. Even though soy lends itself well to research, there are multiple concerns about its future as a source of alternative proteins for human consumption, such as the competition that exists in the use of soy for animal feed formulation, the link between large-scale soy farming and deforestation practices in some countries [43], huge food miles from export, in addition to issues concerning GMOs, allergies, and the preservation and/or valorisation of biodiversity, which have necessitated and warranted a shift from the current over-dependence on soy [3]. Thus, the diversification of the plant protein ingredient portfolio is fundamental to maintaining sustainability and biodiversity and future-proofing the ingredient supply chain, as well as to improving the micronutrient content and overall nutrition profile of the currently marketed products as the plant-based meat alternative sector grows and contributes toward future foods [44,45,46]. Consequently, a lot of emphasis and the efforts of many researchers have focused on generating more palatable meat substitutes from plant protein sources other than soybeans [39,47,48,49].

Depending on the nature and type of plant protein used in the meat alternative, the structural, functional, nutritional, and organoleptic properties of the final product can be manipulated [37,38,50]. The typical protein ingredients used in meat alternatives are either protein isolates or concentrates. Protein isolates are purer fractions with high functionalities, but their processing incurs higher costs and is relatively unsustainable due to the use of more energy and solvents for the extra purification, whereas concentrates have a lower protein content and are somewhat less functional than isolates, although they are better in terms of cost and sustainability. Key properties of plant-based meat alternatives are provided in Figure 1 which are related to the techno-functional characteristics of the plant protein ingredient used (see Section 3), the type of processing to texturize the protein (see Section 4), as well as the other ingredients (e.g., binder, salt, fat, flavour among others) that are added in the final product formulation.

## 3. Techno-Functional Properties and Nutritional Equivalency of Plant Proteins for Meat Alternatives

The techno-functional properties of ingredients play a significant role in the new product development of foods for the future [51]. As the need to search for sustainable protein ingredients continues, the use of proteins of plant origin as a key ingredient in the formulation of meat analogues has emerged [52,53]. In general, based on total dry matter basis, plant protein comprises more than 50% of the plant-based meat formulation and, therefore, acts as one of the key drivers of taste and texture [54]. Thus, the techno-functional properties of plant proteins are crucial for defining their applicability in plant-based meat alternatives. Several studies have already shown a direct relationship between the functional properties of plant protein ingredients and the protein extraction methods applied [55,56]. The basic formulation of the currently available plant-based meats consists of water (50–80%), textured plant proteins (10–25%), nontextured proteins (4–20%), lipids (0–15%), and additives, including flavourings (1–10%) [7,54,57]. A summary of plant protein materials and other ingredients that are used for meat analogue applications is presented in Table 2.

There has been research aimed at understanding how protein functionality can be manipulated to contribute to improving the sensory properties of plant-based meat alternatives [7,58,59] In terms of swelling characteristics, plant proteins were characterized into heat-swelling or cold-swelling, which can be used to formulate meat analogue products targeting specific texture attributes [37].

The extrusion of soy proteins helps to produce texturized plant proteins, and their use in the formulation of meat analogues is well documented [39,60,61,62]. The production of meat alternatives can be achieved using soy protein on its own or, in some cases, by adding wheat gluten [63,64]. The feasibility of using various proteins of plant origin other than soy in food formulations undoubtedly does depend on the techno-functional attributes, which include their solubility, the ability to absorb water and oil, as well as their thermal, gelling, and emulsifying properties, which are all important contributors in defining the quality of the finished product [11,65,66]. According to USDA FAS (2021), the next generation of meat analogues is plant-based meat alternatives using multiple sources of plant-based ingredients, besides soy [67,68], with examples of such proteins being provided in Table 2. Further research is, therefore, required to ensure that hitherto underutilised plant proteins can overcome functional, organoleptic, and techno-economic challenges for replacing soy protein in the plant-based meat alternative formulation. The protein contents of other conventional meat products versus meat alternatives, such as This Isn’t Beef Plant-Based Burgers, which are made from ingredients such as rehydrated, textured pea protein and faba beans, are highlighted in Table 3, and some of the common ingredients that are often used in the formulation of plant-based meat alternatives are presented in Table 2. Soy and pea protein isolates and concentrates tend to have a significantly higher protein content compared to faba beans and lupine (Table 1). However, faba beans still possess a relatively high protein content, which is even higher than defatted soy meal and spray-dried soymilk powder; nevertheless, faba beans have inferior functional properties compared to soy protein. Lupine protein tends to have a much lower protein content (Table 1). Nonetheless, when used as a blend, for instance, with lupine protein concentrate and isolate, there is the possibility for these mixes to be used in the formulation of extruded, texturized meat analogues with fibrous structures [69]. This provides room for the use of protein blends in the formulation of meat alternatives (Table 4) and would allow for underutilised proteins that can sometimes have a lower protein content to still be incorporated into various products.

There is early research showing that blended plant proteins offer promising ingredient opportunities to improve sensory, nutritional, and technological aspects without relying heavily on fortification and additives [70,71]. Protein blends can also optimize overall protein functionality [72]. In addition to the choice of plant protein ingredients, their interaction in various matrices can further modulate their functional properties, which can have a concomitant impact on the structural and sensory characteristics of the meat alternatives [37,38,56]. Therefore, there is a need to understand the intricate complexities that occur in choosing the type of plant proteins, as well as the process they undergo to be able to efficiently exhibit their quality attributes in the formulation of meat alternatives.

One of the major considerations of consumers when purchasing food is the nutritional aspects, which include the nutritional value. The composition of meat alternatives, which includes the total protein, fat, moisture, and carbohydrate content, can impact the nutritional aspects of the food product (Figure 1). In terms of the nutritional value of plant-based meat alternatives, there is a range of nutrients that are present, and a comparison is made to conventional meat [73,74]. Traditional meat is shown to contain relatively high calories and fat, including saturated fat (Table 3), compared to plant-based meat alternatives. Traditional meat does not contain any fibre, whereas plant-based meat alternatives contain some fibre/100 g. The consumption of dietary fibre has been related to a reduction in the incidences of several types of diseases. This is mainly attributed to fibre’s beneficial effects, such as increasing the volume of faecal bulk, the reduction of the intestinal transit time, the modulation of cholesterol, and having an impact on glycaemic levels, in addition to trapping substances (such as mutagenic and carcinogenic agents) that can be dangerous for the human organs, as well as the stimulation of the proliferation of the intestinal flora, among others [75]. Plants also contain various pigments, as well as micronutrients, which if preserved during processing can have a positive impact on health. However, information on the micronutrient composition of plant-based meat alternatives is limited [46]. The protein content does seem to be comparable for both traditional meat and plant-based meat alternatives, while the sodium content seems to be higher in plant-based meat alternatives compared to traditional meat [76]. A high consumption of salt has been related to an increased risk of high blood pressure and is linked with an increased risk of the onset of hypertension and cardiovascular complications [77].

The other issue related to plant-based meat alternatives is the number of additives, as well as the processing that they undergo, with some researchers suggesting that they be classified under ultra-processed foods [78]. Additionally, these meat alternatives tend to be lower in vitamin B12, which contributes to various functions in the human body, such as the development, myelination, and function of the central nervous system, the formation of healthy red blood, and the synthesis of DNA, among others [79], and is predominantly found in animal sources [80]. Hence, people who predominantly follow restrictive, plant-based diets, where meat is completely excluded from the diet, need to consider vitamin B12 supplementation [5]. Nonetheless, a recent study on the Asian population failed to substantiate any superior cardiometabolic health benefits of a diet with plant-based meat alternatives compared to a conventional, meat-based diet [81]. Even though some pros can be seen in the nutritional value of plant-based meat alternatives, there is a need to map the pros and cons of the nutritional values versus the risk benefits before plant-based meat alternatives can become a staple in the diet of many individuals [82].

**Table 2:** Examples of ingredients that are used in the formulation of plant-based meat alternatives, adapted from [69].

Ingredients that Are Used for the Formulation of Meat Analogues	Function	Possible Sources
Colourings	Helps in the achievement of a good appearance and the enhancement of visual appeal.	Annatto, beet juice extract, caramel colours, carotene, cumin, soy leghaemoglobin, malt, and turmeric.
Fat	The enhancement of flavour via the synthesis of volatile and non-volatile flavour constituents. They give the juiciness and tenderness to meat analogues, as well as contribute to the structure formation in meat analogues.	Various oils, such as Canola oil, coconut oil, sunflower oil, corn oil, sesame oil, cocoa butter, olive oil, palm oil, soybean oil, and flaxseed oil.
Flavourings	Contribute towards the enhancement of flavour, as well as act as a preservative for shelf-life extension and mask the flavours of legume proteins.	Various seasonings, natural spices, and herbs, such as basil, fennel, garlic, onion, pepper, and thyme; various precursors, such as amino acids; thiamine; reducing sugars; nucleotides; and yeast extract.
Polysaccharides	Play a key role in the formation of flavour in the presence of amino acids as a result of the Maillard reaction and caramelization occurring in the presence of heat.	Starches and flour, binding agents, and gums, such as methylcellulose, acacia gum, xanthan gum, and carrageenan.
Water	Normally used as a source of hydration for all the other dry ingredients. Water affects the functional qualities, such as viscosity, swelling, emulsification, gelation, and foaming, and also helps with the achievement of juiciness and mouthfeel of the final product and also plays an important role in the determination of the viscosity during the extrusion process.	N/A

**Table 3:** The nutritional information of plant-based meat alternatives in comparison to some traditional/conventional meat.

Product	Calories	Total Fat	Saturated Fat	Fibre	Protein	Sodium
**Beef: serving size 100 g**						
Conventional Minced Beef	332	30 g	11 g	0 g	14 g	67 mg
Beyond Burger Beyond Meat	196	12 g	2.9 g	1.2 g	16 g	300 mg
This Isn’t Beef Plant-Based Burgers	224	15.4 g	5 g	3.0 g	14.3 g	400 mg
Vivera Plant Steaks	200	10 g	4.7 g	4.9 g	18 g	440 mg
**Chicken: serving size 100 g**						
Traditional Chicken Nuggets; Waitrose Frozen Breaded Chicken Breast Chunks	210	8.1 g	1 g	1.1 g	19.8 g	292 mg
Quorn Vegetarian Chicken Style Nuggets	190	8.3 g	0.8 g	4.5 g	9.4 g	480 mg
Tesco Plant Chef No-Chicken Nuggets	230	10.2 g	0.9 g	7.2 g	11.1 g	264 mg

Data collected November 2024. This is based on 100 g of the raw/uncooked products and based on the nutritional label information that can be found on the websites of the product manufacturers. Sodium is calculated by dividing the amount of salt listed on the nutritional label by 2.5.

**Table 4:** Some examples of the HME of plant protein blends/mixes and plant proteins with polysaccharides/sugar for meat analogues.

Protein Type *	Formulation	Key Outcome	Reference
**YPPI + RPC**	50:50	Texturization via HME.	[83]
**SPI + WG** **SPI + PPI** **SPI + RBP** **SPI + FPI** **SPI + SPPI**	70:30	SPI-WG and SPI-PPI exhibited superior fibre.	[70]
**SPI + WG**	70:30, 50:50, 30:70	SPI-WG 50:50 exhibits the best fibrous structure.	[63]
**SPI + WG**	80:20, 35:65, 50:50, 65:35, 20:80	Rheology of SPI- and WG-phases determine structure formation.	[84]
**SPI + WG**	10%, 20%, 30% WG with soy (+oil, starch, salt, pumpkin powder)	30% WG addition exhibited fibrous structures interconnected with much smaller fibres.	[85]
**SPI + PkPC**	90:10, 80:20, 70:30, 60:40 (corn starch was added to all concentrations)	More pumpkin protein produces a less aligned structure.	[86]
**SPI + PPI**	90:10	SPI-PPI gel predicts HME texture, PPI weakened structure.	[87]
**PPI + WG**	100:10, 70:30, 50:50, 30:70	30% WG resulted in the highest fibrous structure.	[88]
**HPC + WG** **HPC + CPC**	90:10, 70:30, 50:50 50:50	90:10 hemp–WG best structure and better sensory with higher hemp fibrous degree.	[61]
**PP + WG**	90:10	PP-CP-WG > PP + WG > PP-SPI-WG; hardness PP-CP-WG > PP + WG ~ PP-SPI-WG; chewiness PPI-CP-WG > PP + WG ~ PPI-SPI-WG fibrous degree PP-CP-WG > PP + WG > PP-SPI-WG; hardness PP-CP-WG > PP + WG ~ PP-SPI- WG; chewiness PP-CP-WG > PP + WG ~ PP-SPI-WG.	[89]
**PP + SPI + WG**	80:10:10		
**PP + CP + WG**	80:10:10		
**PPI + FPC**	93.84:3, 90.84:6, 88.84:8 (3.16% salt added to all)	Noticeable change in the nature of fibre formation due to FPC addition.	[90]
**HPI + PPC** **HPI + SPC** **HPC + PPC** **HPC + SPC**	80:20, 50:50, 20:80	Hemp protein isolate was not able to extrude on its own; extrusion could be performed using hemp protein concentrate and by adding soy or pea proteins.	[91]
**SPI**	Added insoluble dietary fibre (IDF)	IDF facilitates the formation of filamentous structures and enhances the mechanical anisotropy of extrudates.	[92]
**SPI and SPC**	Added inulin	Inulin facilitates the formation of lamellar structures and improves the fibrous structure and protein digestibility.	[93]
**SPI**	Added dietary fibre (DF) from soy	Protein–DF ratio affected extrudate texture, (micro) structure, and protein reactivity.	[94]
**SPI + WG**	Added alginate, xanthan gum, and maltodextrin (1–5% for each)	The polysaccharides increased the water distribution of extrudates by enhancing protein–water interactions through hydrogen bonding.	[95]
**PPI**	Amylopectin and stearic acid	Amylopectin and stearic acid synergistically contributed to improving the fibrous structures and fibrous degree in pea protein extrudate.	[96]
**SPI**	High-acyl gellan gum, low-acyl gellan gum, high-methoxy pectin, low-methoxy pectin, and xanthan (2% for each, with salt)	The addition of hydrocolloids and salts increased the crosslink bonds and structural compactness at the microscopic level and enhanced the fibrous structure.	[97]

* Abbreviations: **YPPI** = yellow pea protein isolate; **RPC** = rapeseed protein concentrate; **SPI** = soy protein isolate; **WG** = wheat gluten; **PPI** = pea protein isolate; **PP** = pea protein; **RBP** = rice bran protein; **FPI** = faba bean protein isolate; **SPPI** = spirulina protein; **PkPC** = pumpkin protein concentrate; **HPC** = hemp protein concentrate; **CPC** = chickpea protein concentrate; **CP** = chickpea protein; **FPC** = flax seed protein concentrate; **HPI** = hemp protein isolate; **PPC** = pea protein concentrate; **SPC** = soy protein concentrate.

## 4. Protein Texturization for Plant-Based Meat Alternatives

Meat has a hierarchical microstructure where individual proteins form fibrils that organise into a fibrous network, and the texture arises from such alignment of fibrous bundles of protein, and this is what the plant protein texturization process aims to mimic [38,98]. To create such a meat-like texture, globular plant protein must unfold, crosslink, and align into highly ordered and distinctive fibrous structures, and for this to happen, extrusion is, so far, the most established and commonly used industrial-scale conversion process [98,99,100,101]. According to GFI’s analysis, 1000 commercial-scale extrusion lines will be required if the plant-based meat alternative market reaches 3% of the total meat production volume (12.5 million metric tons per year) by 2030 [102,103]. This highlights the importance of a quick scale-up of extrusion processes for plant proteins, as well as an opportunity for an increased extrusion throughput capacity in the future, which should also help with bringing down the manufacturing cost.

Extrusion cooking was initially used in the 1970s to produce meat-like products, mainly using soy protein [104]. Ever since, the extrusion of plant-based products, such as meat analogues, has significantly advanced as more research has been carried out to corroborate its use in food processing due to its advantages. On one hand, the extrusion process has been shown to denature undesirable enzymes and anti-nutritional factors, such as tannins, trypsin inhibitors, and phytates, which results in higher digestibility values of proteins [105]. On the other hand, there have been suggestions that the functional properties of plant proteins, such as the emulsification stability, water-holding capacity, and foaming ability, may be improved by extrusion cooking [106]. Extrusion processing can improve these properties but also lead to lipid oxidation and protein denaturation, which can affect the quality and nutritional value of the final product. Extrusion can also enhance flavour and induce protein unfolding, which increases the cleavage site exposure, thereby improving the product’s digestibility [105,107]. Therefore, extrusion has become the most preferred commercial technique for use in the conversion of plant materials into fibrous products [84].

High-moisture extrusion (HME) is one of the commonly used methods in the formulation of meat analogues from plant proteins. HME uses a combination of high shear force, pressure, and temperature with 50–70% moisture to transform the globular plant protein into a fibrous, anisotropic texture [108]. The physicochemical properties of the protein (denaturation temperature, gelling behaviour, surface hydrophobicity, etc.), the characteristics of the protein matrix (e.g., the pH, ionic strength, salt concentration), the HME process variables (e.g., screw speed, melt temperature etc.), and the presence of additional ingredients like fat and/or polysaccharides impact the rheology of the proteinaceous melt and the concomitant fibrous texture formation [109,110]. The phase separation of biopolymers and moisture content during extrusion influences the texture formation as water acts as a plasticizer to influence the biopolymer aggregation and the melt viscosity during HME [111,112]. The phase separation of proteins into a homogenous continuous phase and a dispersed phase during HME is considered an important step in the fibre formation process, and there are two models explaining this mechanistically. Mitchell proposed that the phase separation occurs in the barrel and die head of the extruder [113], whereas there is another theory by Murillo, et al. which proposes spinodal phase separation that occurs under the influence of a temperature gradient [114].

Despite showcasing several advantages of HME technology for producing meat-like textures from plant proteins [115], there is still a lack of systematic understanding of the processing principle applied to plant proteins [63]. The mechanistic understanding of plant protein texturization under various HME process conditions has, so far, been poorly understood. There are studies on HME using soy [116,117,118], pea [119], hemp [39], and faba [120,121]. It was shown that a soy protein and wheat gluten blend form a dispersed bi-phasic network under HME and its rheological properties, and the water absorption capacity of the protein largely determines the structure formation process [84,122]. Similarly, the co-extrusion of proteins from pea and gluten [50,95], as well as various mixes of soy, pea, and gluten proteins [89], demonstrated fibrous texture formation. There are three pivotal factors through which gluten facilitates texturization during the co-extrusion process—(a) the capacity to create phase separation, (b) having a highly elastic behaviour and promoting the right water network, and (c) the presence of a cysteine-rich protein forms a disulfide bond with the other protein phase, which leads to the formation of the viscoelastic network. Further in-depth studies are required to elucidate the mechanisms behind the network-forming behaviour of other underutilised plant proteins during HME, which should enable more precise control over the texture creation of plant-based meat alternative products [123]. Moreover, studies on tackling the flavour challenges of extruded plant proteins are rather limited [89,95] compared to the studies performed for textural improvement, which indicates a lack of scientific insight into the texture–flavour inter-relationship.

There is a growing interest in exploring the HME of plant protein blends, as well as in using a combination of plant proteins and polysaccharides to develop meat-like fibrous structures. Examples of plant protein blends and carbohydrate mixes that have been extruded into meat analogues are presented in Table 4. There have been interesting observations of when proteins are mixed with other proteins or carbohydrates. For example, when used as a blend, for instance, of lupine protein concentrate and isolate, there was the possibility for these mixes to be used in the formulation of extruded texturized meat analogues with fibrous structures [69]. A typical protein blend consists of soy and wheat gluten, providing a highly digestible, indispensable amino acid score and superior gelation abilities. A 50/50 blend of soy protein and wheat gluten was shown to result in the best fibrous structures, whereas extrudates prepared by either soy protein concentrate or wheat gluten did not contain any clear fibrous structures [63]. Zhang and coworkers reported that hydrogen and disulfide bonds played a major role in the formation of fibrous structures over extrusion. In their work, they showed that a transformation of disulfide bonds from the intramolecular to intermolecular mode and the transformation from an α-helix to a β-sheet structure while maintaining an “ideal balance” of these protein structures played a significant role in the formation of the fibre structure [63]. Texturized plant protein extrudates have also been successfully developed from rapeseed protein concentrates and yellow pea proteins at 50:50 ratios [83], as well as from hemp protein concentrates and wheat gluten and chickpea protein concentrate at various concentrations, as summarised in Table 4. The exploration of the extrudability of plant protein blends other than soy protein is a plausible idea as understanding their ability to be used in the formulation of meat analogues would allow for underutilised proteins that can sometimes have a lower protein content to still be incorporated into various products, as aforementioned. This provides room for the use of protein blends being used in the formulation of meat alternatives.

Research has shown, so far, that blending different plant proteins can result in meat analogues with better nutritional, textural, and sensory properties [70,124]. For plant proteins like soy protein to be applied as animal protein analogues, one key step is crosslinking, which has been shown to result in some fundamental changes of characteristic quality-related properties, such as rheological qualities. For instance, the textural profiles and structures of plant-based meat alternatives were found to be related to their viscoelastic properties and were strongly affected by the extent of crosslinking between protein molecules [125]. Some studies have shown that the disulphide–hydrogen interactions that occur in wheat gluten result in an increase in protein crosslinking, and this has the potential to modulate the formation of fibrous structures of meat analogues [85,126]. The incorporation of a higher pea protein concentration into plant-based protein nuggets was shown to result in an increase of both storage and loss moduli, indicating an increase in the protein gel strength, suggesting an increase in the crosslink networks [125].

Extrusion studies using soy, wheat gluten, and pea proteins demonstrated that cold-swelling protein or a higher ratio of cold-swelling protein to heat-swelling protein in a protein blend led to an increased degree of crosslinking and greater porosity, which promoted the higher softness of texturized vegetable protein [37]. During the extrusion process, proteins are subjected to high pressures and temperatures, which result in their denaturation and, consequently, the loss of their tertiary or even secondary structure [63]. The denatured proteins will usually realign in the direction of flow as they move through the screw, and this exposes binding sites that allow for a new way of crosslinking the proteins. This crosslinking is responsible for the texturization and transformation of globular plant proteins into structures that more closely resemble the fibrous and laminar construction of meat [127], although the sensorial and textural attributes of these products are often thought to be inferior to meat. To generate a better texture, the manipulation of ingredients that have different functionalities could be a critical step for plant-based meat analogues to improve their sensory attributes. When protein was mixed with polysaccharides, such as dietary fibre, edible gums, starches, etc., Tang and co-workers showed that the mixtures underwent intricate conformational changes and helped fill the gaps in the protein network via covalent, as well as non-covalent, interactions under extrusion conditions, thereby forming a complex, three-dimensional network to enhance the stability of the overall structure of plant protein extrudates [56]. Marczak and Mendes have suggested that dietary fibres exhibit interesting opportunities for modelling and the improvement of texture and other functional properties of meat alternatives [128]. Polysaccharides have also been shown to play an important role in controlling the formation of protein aggregates. The aggregation of soy protein is suppressed when gum Arabic was added to the matrix, which was mainly attributed to protein–polysaccharide electrostatic interaction [41]. This finding paves the way for future research to be carried out to evaluate how protein–protein and protein–polysaccharide interactions affect the generation of networks that would contribute to meat-like structure formation.

During the formulation of a meaty texture, various other techniques can also be employed that vary from shear cell technology [104] to recent advances in 3D printing [129], as well as more exploratory techniques, such as electrospinning [130]. In electrospinning, a high voltage is applied to protein solutions pushed through a spinneret, forming edible submicron and even nano-sized fibres. This technology has been explored for proteins, including pea [131], bean [132], and zein [133]. Overall, the application of spinning technology for plant protein texturization can be viewed much the same way as 3D printing in terms of structure formation, but the process throughput is, so far, too low for any commercial viability. The use of 3D printing [134] has demonstrated an unprecedented capacity to fabricate food products with intricate structures and reduced material costs and energy consumption [135]. Miller and colleagues have looked at 3D printing and the consumer acceptance of alternative meats. In their work, they highlight the major advantage of 3D printing as being the ease with which ingredients can be customised in terms of their colour, flavour, and nutritional components, as well as the customisable shape and texture of the final products [134]. These aspects are critical to the formulation of meat alternatives as they are key to consumers’ acceptance of and preference for these products. However, it is important to note the fact that there are currently limited types of materials available for 3D printing, the throughput is still a challenge, and consumers remain unfamiliar with this novel technology. In addition to the fact that building acceptance and trust is highly important in order for consumers to be willing to purchase and consume 3D-printed, plant-based meat alternatives, there is a need for consumer awareness and sensitisation on the pros and cons of such emerging technologies. This can be achieved through transparency in all aspects of the design and creation of these new products.

The other texturizing technique that has been used in the formulation of meat alternatives is freeze structuring or freeze alignment. This technique involves the freezing of an aqueous solution or a slurry of proteins to generate the structure [84,136]. The removal of heat from a slurry that is well mixed does lead to the formation of an isotropic structure. However, the unidirectional removal of heat without mixing the slurry can result in the alignment of ice crystal needles that yield anisotropic structures. The directional freezing of protein slurries has been studied for the structuring of meat, fish, and plant [137,138]. The freezing temperature and rate are key in tailoring the size of the ice crystal needles, and the good solubility of the proteins before freezing is vital to obtain distinct fibrous products [139]. The frozen products are subsequently dried without melting the ice crystals. The drying can be achieved, for example, by freeze drying, which results in the formation of a porous microstructure that has a sheet-like parallel orientation of the proteins [84]. These sheets are connected, forming a cohesive product that is fibrous in nature that can be a good scaffold for the formation of meaty structures using alternative proteins. The lack of solubility of plant-based protein can, however, limit the use of freeze structing or free alignment in the formulation of meat alternatives. This is particularly of concern depending on how the proteins are extracted. Conventional wet extraction for producing plant protein concentrates and isolates requires the removal of water to increase the concentration of proteins. In most cases, spray drying and freeze drying are used to dry the protein, but this has been shown to result in structural changes to the proteins that have the potential to result in a reduction in protein solubility [1]. An emerging and energy-efficient protein separation technology using air-classification-based dry fractionation [140,141] should be further investigated for producing optimal protein ingredients to be used for the freeze-structuring method.

## 5. Environmental Sustainability of Plant-Based Alternatives to Meat Products

**Figure 2:**
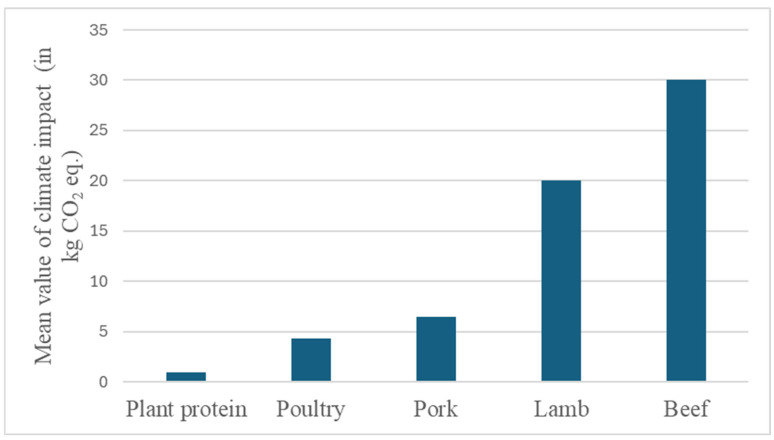
Comparison of the climate impact per 100 g of the plant-based and animal protein contents of the final product (Data used from [144,145]).

The environmental impact of meat alternatives has been and continues to be studied. For instance, a recent systematic review screened 34 publications evaluating 135 plant-based meat alternative products in high-income countries with 53 animal-based product comparators and reported a more than 70% reduction in greenhouse gas emission, land use, and water usage for most products when shifting from animal-based to plant-based [142]. A median climate impact of plant-based meat alternatives was estimated to be 1.7 kg CO_2_ eq./kg of product, with a variation of 0.67 to 2.54 kg CO_2_ eq./kg of product [143,144]. The climate impact per protein content of the product was estimated to be 0.94 kg CO_2_ eq. per 100 g of plant protein, with a variation of -0.63 to 1.50 kg CO_2_ eq. per 100 g of protein [144,145], which, considering the mean values, is still ~4.5 times less than poultry, ~6.9 times less than pork, ~21 times less than lamb, and ~32 times less than beef, as illustrated in Figure 2, which shows a comparison of the climate impact per 100 g of the plant-based and animal protein contents of the final product. To evaluate the potential environmental sustainability aspects that are related to the use of plant-based alternatives to meat products, one needs to consider the necessary steps that are required in the processing and purification of the proteins, as these are the key determinants of the chemicals and resources that are required in addition to the energy requirements. When considering the potential environmental sustainability gains, pulses require very little processing before being used and, hence, could be deemed to have more environmentally sustainable alternatives to meat products. However, the potential gains in sustainability are shown to become uncertain (and in some cases, probably low) for alternatives that require a lot of processing [146]. This would apply to plant-based alternatives that are made using highly purified and processed ingredients, such as protein isolates or protein-rich products derived from sources such as algae [147].

## 6. Market, Innovation, and Supply Chain of Plant-Based Meat Alternatives

The plant-based meat alternatives reached global retail sales of USD 5.6 billion in 2021 [148] and showed modest growth with an 8% retail sales increase in 2022. The total market size of plant-based meat alternatives is estimated to be USD 33.99 billion by 2027, which is only 2.5% of the global meat sector [11]. According to forecasts by Bloomberg Intelligence (New York, NY, USA) and Credit Suisse (Zürich, Switzerland), the global sales of plant-based meat alternatives could reach anywhere between USD 74–118 and USD 88–263 billion, respectively, by 2030 [148]. The acceleration of meat alternative technologies is hugely driven by private investments ranging from venture capitalists to large food corporates across FMCGs like Unilever (London, UK) and Nestle (Vevey, Switzerland), food ingredient producers like ADM (Chicago, IL, USA), Cargill (Hong Kong), Bunge (Chesterfield, MI, USA), etc., as well as big meat producers like JBS (São Paulo, Brazil) and Tyson (Springdale, AR, USA) [149]. In 2023, Denmark published the world’s first national action plan for plant-based foods, which is a follow-up of the EUR 168 million public funding announced in 2021. Moreover, during 2021–2024, several countries, like Germany, Catalonia, Netherlands, the UK, Singapore, and Israel, committed large amounts of public funding for alternative protein R&D [150,151]. Despite this large-scale public investment, since 2021, the plant-based meat market has shown signs of stagnation and some consolidation, with venture capital funding for early-stage start-ups drying up. Examples of recent consolidation include the acquisition of brands like Meatless Farm (Leeds, UK) and Clive’s Purely Plants (Dartmouth, UK) by the Vegan Food Group (VFC, York, UK); Livekindly Collectives (New York, NY, USA), which was acquired by Alpha Foods (Glendale, CA, USA) in the US; as well as All G Foods (Sydney, Australia), which separated from plant-based meat brand Love Buds (Denver, CO, USA) to merge with Fenn Foods’ vEEF brand and form the Aussie Plant-Based Co. (Melbourne, Australia).

According to the GFI report [102], the projected ingredient requirements needed for a scenario to produce ~25 million tonnes of plant-based meat alternatives in 2030 while mirroring the ingredient composition of top products in today’s market (62% wheat- and soy-based) would require using 2% of the global wheat and soy production. This may need three times the current global supply of soy protein concentrate, which points towards the capacity expansion of ingredient processing. There arises a need to research the possibility of incorporating other plant-based protein sources, such as pea proteins, in various formulations. However, pea protein may pose more problems, because, in the same scenario, the plant-based meat alternative industry could require ten times the projected global supply of pea protein and 34% of pea production overall. Because of its relatively low protein content, such an enormous expansion of pea protein production would lead to large volumes of byproduct, namely starch, and this would necessitate the need to think of what to do with the starch to avoid it making its way to landfill or needing ways to get rid of the waste streams. Thus, the future market growth of plant-based meat alternatives does require critical insights into supply chain trade-offs and concomitant innovation to diversify protein ingredients.

The techno-economics of plant protein ingredient selection, as well as economy of scale, process throughput, and energy usage in production, should significantly influence the manufacturing scale-up opportunity, as well as drive the overall sustainability benefits. According to a recent Deloitte study [152], supplier relationship management and technical competency are critical to addressing complicated risk management, developing integrated procurement processes, boosting efficiency, and reducing price volatility in the supply chain of plant-based meat alternatives. A recent study also showed the importance of downstream value chains for the successful market penetration of plant-based meat, which includes distribution, the marketing/promotion of health benefits, and retailer relationships [145].

Most start-ups and micro-SMEs across the globe have driven the technological innovation in the plant-based meat alternative sector [153], but their R&D operations, investment caps, and business models are very different from big food companies. As such, the plant-based meat sector requires an inter-disciplinary ecosystem approach [153] to navigate complex regulatory pathways, deliver science-led policy decisions, understand the influence of social transitions [154], and also evaluate the role of institutional factors, including the drive from multinational companies in the sector development [155]. This provides numerous opportunities for industry–academia collaboration, which should play a critical role in such innovation growth by fostering knowledge exchange.

## 7. Challenges and Opportunities for Plant-Based Meat Alternatives

**Figure 3:**
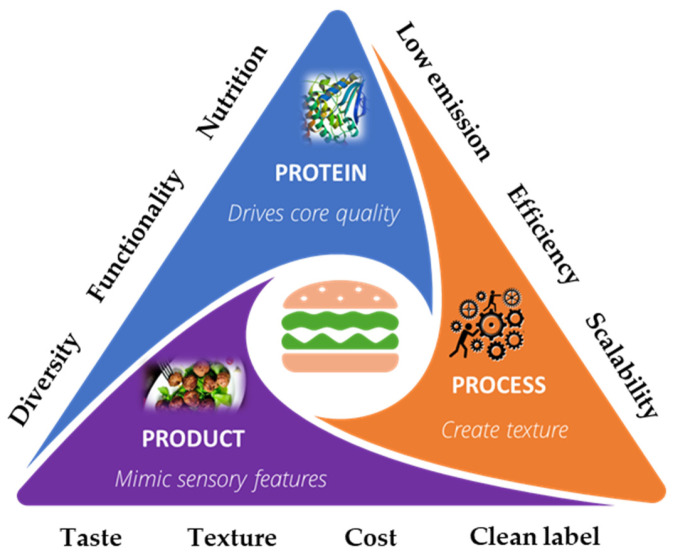
The technological landscape of plant-based meat alternatives to address formulation challenges.

Despite the growing technological innovation in plant protein texturization to mimic meat’s texture [156], there are still protein ingredient-related challenges and product-related quality gaps to be overcome to improve the consumer acceptance of plant-based meat alternatives. A summary of the technological landscape is presented in Figure 3.

To sustainably mitigate the growing demand for meat, there are wide varieties of plant-based meat alternatives in terms of the food category, ingredient usage, and/or claims [157]. Nevertheless, to the best of our knowledge, there are no clean-label, plant-based formulations on the market today that can ensure a meat-like palatability and match the nutrition profile of the meat. The other key challenge includes the cost of plant-based meat alternatives [5,88]. The reduced demand for plant-based meat in 2022, particularly in the US market, as measured from the sales volume of companies like Beyond Meat and Impossible Foods, was due to high costs and inflation coupled with market challenges [148]. Thus, the retail price of meat alternatives is a critical factor for wider consumer acceptability and influences repeat purchases. According to Nielsen’s data, on average, plant-based meat is twice as expensive as beef, more than four times as expensive as chicken, and more than three times as expensive as pork on a per-pound basis [158]. The gap in organoleptic properties of plant-based meats, particularly their texture and taste, compared to conventional meat products, is a major obstacle in consumer acceptance. The different ingredients that are used in the formulation of meat alternatives (Table 2) grant different textural functionalities and sensorial attributes. Economically scaling up the manufacturing process presents a significant challenge by maintaining the performance of formulations produced at the laboratory scale whilst producing at larger scales. A techno-economic analysis is essential to predict the costs of processes at different scales and identify key roadblocks, as well as to improve the processes’ efficiency. Moreover, in addition to technology gaps, the other major barriers to marketing the expansion of plant-based meat alternatives are consumer food habits, for example, the adaptability and familiarity aspects, which include the lack of any previous experience with such products [8]. This may become an impediment to plant-based foods’ incorporation into many households as a staple food but would not be for those who definitely have no other option than to buy these alternatives. A study by Yule and Cummings also showed the presence of incongruence in the marketing communication of plant-based meat, which fails to win over conservative consumers [158]. The other issue is the processing of these meat alternatives. Given the ingredients and the processing, these alternatives have been suggested to be under the category of ultra-processed foods (UPFs), as aforementioned. UPFs have been linked to other comorbidities and health outcomes, such as cardiovascular diseases, obesity, and type 2 diabetes. According to a recent survey by the EIT Food Consumer Observatory, 54% of European consumers feel discouraged from buying plant-based meat from supermarkets amid concerns these could be classified as UPFs [159]. However, there is a lack of consensus among experts on the NOVA-classification-system-based guidance on UPFs because it is not based on healthiness but solely on the level of processing [160]. It is, therefore, important to understand the relationship between plant protein functions and their impact on processing because such insights can help re-optimise the organoleptic and functional properties of plant proteins using mild processing technologies [31]. Finally, the issue to do with labelling continues to be a stumbling block, with some countries even banning the sale of products that are labelled as meat but are not of animal origin [161]. These barriers need to be removed to allow for the widespread inclusion of plant-based meat alternatives in the day-to-day diet currently and in the formulation of future foods that will include underutilised and underexploited, as well as novel, proteins of plant origin.

## 8. Conclusions

Our livestock-reliant food system operates unsustainably, but it is also unrealistic to expect a sudden change in global food production by introducing meat analogues made of various alternative plant protein sources without guaranteeing their cost competitiveness, quality, and safety, as well as ensuring their health benefits, which contribute to the acceptance of future foods. The introduction of post-launch monitoring, including nutravigilance, should be broadly implemented. Further research is required to ensure that hitherto underutilised plant proteins can overcome functional, nutritional, organoleptic, and techno-economic challenges for replacing soy protein in plant-based meat alternative formulation. For the further growth of the plant-based meat sector as future foods, it is essential to achieve taste and texture parity with traditional meat on a cost-neutral basis while delivering nutritional equivalency and clean-label products via low-emission processing. Various consumer studies on plant-based meat alternatives and market research post-2021 clearly showcase that technological innovation may not be the only unlocking factor for sectorial growth. The other key determinants are consumer insights in terms of the varied nature of the adoption of plant-based meat across countries, as well as the differential socioeconomics of the protein transition. Thus, novel technologies should be scrutinised through the lens of consumer adaptability and affordability to reduce the risk of innovation, thereby enabling market growth.

## Figures and Tables

**Table 1:** A summary of some protein ingredients that are used for meat analogue applications (adapted from the work of Munialo and Vriesekoop [3]).

Material Composition (%*w*/*w*)	Protein Ingredient	Functionality	The Type of Application in Meat Analogues		
			Structuring Process	Role	Products
~70% protein	Soy concentrate	Possesses excellent texturization properties.	Extrusion and shear cell technology	As a binding ingredient, a source of protein, and in the enhancement of texture.	Most burger patties, minced meat analogues, muscle-type products, and plant-based sausages.
~90% protein	Soy isolates extracted by the use of alkaline/acid precipitation	Possess excellent gelation and emulsification potential, as well as good solubility propensity.	Extrusion, shear cell technology, spinning, and freeze structuring.	Used as the base for the formulation of fat substitutes, as a binder, as a protein source, emulsifier, and for texture enhancement.	Most burger patties, minced meat analogues, and plant-based sausages.
~90% protein that is mainly denatured due to heat treatment	Soy isolates that are extracted from soy that has been heat treated or toasted isolate	There is a general decrease in solubility, an increase in water-holding capacity, and good gelling propensity.	Extrusion, shear cell	Used as a protein source, texture, binder, a base for fat substitutes.	Used in the formulation of burger patties, minced meat analogues, and plant-based sausages.
~43–56% protein, ~0.5–9% fat, ~3–7% crude fibre, and >30% total carbohydrate	Defatted soy flour/meal	They display good water-binding capacity and fat-retention properties.	Extrusion	Used as a binding ingredient and in texture modification.	Used in the formulation of burger patties, minced meat analogues, in the production of muscle-type products, and in plant-based sausages.
>45% protein, ~30% fat	Spray-dried soymilk powder	Tend to be highly soluble and possess good emulsification properties.	Mainly used in freeze structuring	Used as an emulsifier and in texture modification.	Used in the production of tofu and Yuba.
~85% protein	Pea isolates	Possesses good water- and fat-binding properties, emulsification, and results in a firm texture after thermal/heat processing.	Used in extrusion, shear cell technology and in spinning	Used as a binder, emulsifier, and texture enhancement.	Used in the formulation of burger patties, the production of minced-meat analogues, the formulation of muscle-type products, and plant-based sausages.
75–80% protein, 15–17% carbohydrates, 5–8% fat	Wheat gluten isolate	Possess good binding ability, low solubility, is involved in the formation of dough, and has a high crosslinking capacity via S-S bridges.	Used in extrusion, shear cell technology	Used in functions that require adhesion properties and in the enhancement of the textural properties of meat analogues.	Used in the formulation of burger patties and in the formulation of muscle-type products.
56% protein	Dry, fractionated faba	Possesses good water-binding properties, gelation, and results in a firm texture after thermal/heat processing.	Used in high-moisture extrusion	Used in the enhancement of textural properties.	Used in the formulation of burger patties, the production of minced meat analogues, the formulation of muscle-type products, and plant-based sausages
~37% protein	30% native lupine flour blended with 70% different blend ratios of lupine protein concentrate and isolate	Possesses a reduced water-absorption capacity and gelation and results in a firm texture after thermal/heat processing.	Used in extrusion	Used in the enhancement of textural properties.	Used in the fabrication of texturized meat analogues with fibrous structures.

## Data Availability

No data were used for the research described in the article.
